# Biomechanical adaptations during exhaustive runs at 90 to 120% of peak aerobic speed

**DOI:** 10.1038/s41598-023-35345-8

**Published:** 2023-05-22

**Authors:** Aurélien Patoz, Thomas Blokker, Nicola Pedrani, Romain Spicher, Fabio Borrani, Davide Malatesta

**Affiliations:** 1 Institute of Sport Sciences, University of Lausanne, 1015 Lausanne, Switzerland; 2 Research and Development Department, Volodalen Swiss Sport Lab, Aigle, Switzerland

**Keywords:** Health care, Medical research

## Abstract

The aim of this study was to examine how running biomechanics (spatiotemporal and kinetic variables) adapt with exhaustion during treadmill runs at 90, 100, 110, and 120% of the peak aerobic speed (PS) of a maximal incremental aerobic test. Thirteen male runners performed a maximal incremental aerobic test on an instrumented treadmill to determine their PS. Biomechanical variables were evaluated at the start, mid, and end of each run until volitional exhaustion. The change of running biomechanics with fatigue was similar among the four tested speeds. Duty factor and contact and propulsion times increased with exhaustion (*P* ≤ 0.004; *F* ≥ 10.32) while flight time decreased (*P* = 0.02; *F* = 6.67) and stride frequency stayed unchanged (*P* = 0.97; *F* = 0.00). A decrease in vertical and propulsive peak forces were obtained with exhaustion (*P* ≤ 0.002; *F* ≥ 11.52). There was no change in the impact peak with exhaustion (*P* = 0.41; *F* = 1.05). For runners showing impact peaks, the number of impact peaks increased (*P* ≤ 0.04; ${\upchi }^{2}$ ≥ 6.40) together with the vertical loading rate (*P* = 0.005; *F* = 9.61). No changes in total, external, and internal positive mechanical work was reported with exhaustion (*P* ≥ 0.12; *F* ≤ 2.32). Results suggest a tendency towards a “smoother” vertical and horizontal running pattern with exhaustion. A smoother running pattern refers to the development of protective adjustments, leading to a reduction of the load applied to the musculoskeletal system at each running step. This transition seemed continuous between the start and end of the running trials and could be adopted by the runners to decrease the muscle force level during the propulsion phase. Despite these changes with exhaustion, there were no changes in either gesture speed (no alteration of stride frequency) or positive mechanical work, advocating that runners unconsciously organize themselves to maintain a constant whole-body mechanical work output.

## Introduction

Running is defined as a cyclic alternation of lower limb support and flight phases, where the human body must adopt a specific running pattern to move quickly and efficiently. However, within the time course of a prolonged run, the running biomechanics alter due to the appearance of fatigue^1–15^. For instance, several authors suggested the transition towards a smoother vertical running pattern during an ultra-trail^9,10,14,15^. Such smoother vertical running pattern was characterized by a lower peak vertical force (${F}_{v,\text{max}}$) and vertical loading rate as well as a higher stride frequency and duty factor associated with a lower flight time (${t}_{f}$)^10^. These studies suggested that in a fatigued state, protective adjustments developed, leading to a reduction of the load applied to the musculoskeletal system at each running step, including impact shock. Hence, these altered running patterns may have an implication for injury risks and performance during long-run trials^16^. In fact, even if conflictual^17,18^, it has been reported some associations between biomechanical spatiotemporal and kinetic variables and running-related injury (see review for details^17^). For the spatiotemporal variables, lower stride frequency, shorter ground contact time (${t}_{c}$), lower duty factor, and longer step length are reported to be associated with greater injury risks. Moreover, greater vertical impact peak (${F}_{v,\text{impact}}$), vertical loading rate, and peak braking force (i.e., running kinetic variables) are shown to be associated with these increased injury risks^17^. Therefore, it seems relevant to investigate the effect of exhaustive run on the running pattern to prevent running-related injury, optimize training, and improve running performance.

Running biomechanics during exhaustive runs at constant running speed close to the 10-km race pace^7,8^, near the speed associated with maximal oxygen uptake (${s}_{{\dot{\text{V}}}{{\text{O}}}_{2}\text{max}}$; i.e., 90–95% ${s}_{{\dot{\text{V}}}{{\text{O}}}_{2}\text{max}}$^1–4,6,13^) or at ${s}_{{\dot{\text{V}}}{{\text{O}}}_{2}\text{max}}$^5^ has been investigated either on an indoor track^13^ or on a treadmill^1–8^. These studies reported conflicting evidence with respect to changes in running spatiotemporal parameters due to exhaustion. Both a decrease^1,7^ and an increase^4–6,13^ of ${t}_{c}$ was reported through the exhaustive run, and similarly for ${t}_{f}$ [decrease^5,6,13^; increase^4,7^]. As for stride frequency, Hayes and Caplan^5^ reported no change with exhaustion while other authors reported either a decrease^1,2,4,8^ or an increase^13^ with fatigue. Similarly, conflicting results are found for kinetic variables during exhaustive running. In fact, Hanley and Mohan^7^ showed a decrease in ${F}_{v,\text{max}}$ and vertical impulse while ${F}_{v,\text{impact}},$ and vertical loading rate did not change between the start and end of the 10-km run. Likewise, Rabita et al.^13^ observed a decrease in ${F}_{v,\text{max}}$ but no change in vertical stiffness [i.e., the ratio of ${F}_{v,\text{max}}$ over the vertical displacement of the whole-body center of mass (COM) during ${t}_{c}$] characterizing the elastic property of the lower limb spring according to the spring-mass model used to describe the mechanics of running in humans^19,20^. In fact, biomechanically, during running, kinetic and gravitational potential energies are in phase and at their minimum in the middle of the foot contact time when the COM trajectory changes from a downward to an upward displacement (see review for detail^21^). The total positive mechanical work performed during running is composed of two components: the positive external mechanical work needed to raise and accelerate COM within the environment (i.e., the sum of positive increments of potential and kinetic energies of COM^22^) and the positive internal work performed to accelerate the limbs with respect to COM (i.e., the sum of increments of limbs’ kinetic energies^23^). The assessment of the positive mechanical work concomitantly with the energy cost of running is pivotal to determine the apparent mechanical efficiency of running (i.e., the ratio between the total mechanical work and energy cost) and their changes with exhaustion and fatigue. The positive external mechanical work was shown to either increase with exhaustion, due to an increase of positive kinetic work but no change in positive potential work^2^, or to be constant throughout the running trial, with no change in positive potential and kinetic works^4^ or with an increase in positive potential work and a decrease in positive kinetic work^1^.

None of these previous studies^1–8,13^ reported the previously enumerated spatiotemporal and kinetic variables altogether. Additionally, only two studies investigated the ground reaction force and associated variables^7,13^. However, reporting all these variables together could allow to better understand the running pattern, which is a global and dynamic system^24,25^. Moreover, changes of running biomechanics during exhaustive runs at speeds above ${s}_{{\dot{\text{V}}}{{\text{O}}}_{2}\text{max}}$ has not been investigated so far. The understanding of the effect of exhaustion on running biomechanics close to and above ${s}_{{\dot{\text{V}}}{{\text{O}}}_{2}\text{max}}$ might be useful as many races and high-intensity intermittent training sessions are run at these speeds to prevent running-related injury, optimize training, and improve running performance in these specific conditions.

Hence, the purpose of the present study was to examine how running biomechanics (spatiotemporal and kinetic variables) adapts with exhaustion during treadmill runs at speeds corresponding to 90, 100, 110, and 120% of the peak aerobic speed (PS; speed before failure/exhaustion) of a maximal incremental aerobic test. Throughout the time course of the exhaustive runs, we hypothesized that (1) ${t}_{c}$ and ${t}_{f}$ would increase and decrease, respectively, while stride frequency would decrease with fatigue; and (2) ${F}_{v,\text{max}}$ would decrease with no change in vertical loading rate and stiffness. Finally, we also explored the changes in positive internal, external, and total mechanical work with exhaustion because of their link with energy cost and apparent mechanical efficiency of running.

## Materials and methods

### Participants

Thirteen male recreational runners gave written informed consent to participate in the present experiment (age: 25.7 ± 4.4 years; height: 179 ± 5 cm; body mass: 68 ± 5 kg; ${{\dot{\text{V}}}{{\text{O}}}_{2}\text{max}}$: 64.2 ± 4.2 ml/min/kg; PS: 5.3 ± 0.3 m/s). For study inclusion, participants were required to be in good self-reported general health with no symptoms of cardiovascular disease or major coronary risk factors, no current or recent lower-extremity injury that could prevent them from giving 100% of their capacity during the test, and to meet a certain level of running performance. More specifically, runners were required to have a PS greater or equal to 4.44 m/s (16 km/h). Such threshold allowed experimental testing on a fairly homogeneous group. The study protocol was approved by the institutional review board named ethics committee of the Vaud canton (ethics cantonal commission for research on human beings CER-VD 2018-01814) and adhered to the latest Declaration of Helsinki of the World Medical Association.

### Experimental procedure

Each participant completed five experimental sessions in the laboratory interspersed by at least two days. All participants were advised to avoid strenuous exercise the day before a test but to maintain their usual training program otherwise. The rate of perceived exertion scale (RPE; scale: 6–20)^26^ was used right after the end of an exercise to obtain the perception of effort given by the participants during the test (all participants were familiar with using RPE). No information about the timings or running speed was given to any participant, who were strongly encouraged, during any of the five experimental sessions. All participants were familiar with running on a treadmill and wore their own running shoes during testing.

During the first session, participants completed a maximal incremental aerobic test on an instrumented treadmill (Arsalis T150–FMT-MED, Louvain-la-Neuve, Belgium). This test consisted of a 10-min warm-up at 2.78 m/s followed by an incremental increase in the running speed of 0.28 m/s every 2 min until exhaustion^27^. This test was used to determine the PS of the maximal incremental aerobic test of each participant. PS is defined as the running speed of the last fully completed increment (${s}_{\text{last}-\text{inc}}$) plus the percentage of time spent in the following uncompleted increment ($\alpha$) multiplied by the running speed increment (*∆s* = 0.28 m/s)^28^: $\text{PS}={s}_{\text{last}-\text{inc}}+\alpha \Delta s.$

The other four tests were performed in a randomized order and consisted of exhaustive runs at a given percentage of the participant’s PS (90, 100, 110, 120%). These tests were as follows: after a 10-min warm-up at 2.78 m/s and a 5-min rest period, the running speed was increased to a given percentage of PS, and the participant had to maintain the pace until volitional exhaustion. Once participants indicated that they were unable to sustain the exercise intensity, the treadmill was smoothly stopped in ~ 5 s. The time to exhaustion was collected for each of the four sessions. Ground reaction forces (1000 Hz) were collected using the force plate embedded into the treadmill and during the last 30 s of each minute passed in each of the four exhaustive runs. Forces were subsequently low-pass filtered at 20 Hz using a fifth-order bidirectional Butterworth filter. From these data, ten successive strides were selected and chosen to be at the closest to the start, mid, and end of each exhaustive run according to the time at which ground reaction forces were collected. The timings of the strides selected at start, mid, and end are reported in Table 1.

**Table 1:** Means ± standard deviations of the time to exhaustion corresponding to the four exhaustive runs performed at 90, 100, 110, and 120% of the participant’s peak aerobic speed (PS) as well as the timing of the first of the ten consecutive strides selected at the mid and end of the exhaustive runs (the timing of the first of the ten consecutive strides selected at the start of the exhaustive run was always 0.5 min) and the rate of perceived exertion reported right after the end of the exercise (RPE; scale: 6–20).

Running speed (%PS)	90	100	110	120
Time to exhaustion (min)	15.25 ± 2.50	5.68 ± 1.10	2.65 ± 0.78	1.58 ± 0.49
Mid (min)	7.39 ± 1.37	2.68 ± 0.54	1.28 ± 0.40	0.73 ± 0.25
End (min)	14.57 ± 2.61	5.18 ± 1.10	2.33 ± 0.78	1.26 ± 0.56
RPE	18.8 ± 0.9	19.1 ± 0.9	19.1 ± 0.6	18.9 ± 1.0

### Data analysis

#### Temporal variables

Foot-strike and toe-off events were detected by applying a 20 N threshold to the vertical ground reaction force^29^ while mid-stance event was placed at the instant where the fore-aft ground reaction force changed from negative to positive. ${t}_{c}$ and ${t}_{f}$ were defined as the time from foot-strike to toe-off of the same foot and from toe-off of one foot to foot-strike of the contralateral foot, respectively. Swing time (${t}_{s}$) was defined as the time from toe-off to foot-strike of the same foot. ${t}_{c}$ and ${t}_{s}$ were further used to compute stride frequency (SF) and duty factor (DF)^30,31^:

1 $${\text{SF}} = \left( {t_{c} + t_{s} } \right)^{{ - 1}} ,$$

2 $$\text{DF}= {{t}_{c}\cdot ({t}_{c}+{t}_{s})}^{-1}.$$

Braking and propulsive times were given as the time from foot-strike to mid-stance of the same foot and mid-stance to toe-off of the same foot, respectively.

#### Running kinetics

${F}_{v,\text{max}}$ And ${F}_{v,\text{impact}}$ (given as a local maximum on vertical ground reaction force between foot-strike and the timepoint corresponding to ${F}_{v,\text{max}}$, if any) together with the corresponding counts of ${F}_{v,\text{impact}}$ (number of vertical impact peaks) were characterized. More precisely, for a given running step, ${F}_{v,\text{impact}}$ was given by the value at the first timepoint for which the derivative of the vertical ground reaction force became negative. In addition, braking and propulsive peak of the fore-aft ground reaction force during braking and propulsive time, respectively, were computed. Furthermore, vertical loading rate was calculated as the slope of the vertical force between 20 and 80% of time between foot-strike and 15% of ${t}_{c}$^32^. Braking loading rate was obtained as the ratio of the braking peak of the fore-aft ground reaction force (which is negative) over the time from foot-strike to this peak, leading to a negative rate of loading. Propulsive loading rate was obtained as the ratio of the propulsive peak of the fore-aft ground reaction force over the time from mid-stance to this peak. Besides, vertical, braking, and propulsive impulses, i.e., the integral of the vertical ground reaction force during ${t}_{c}$ and of the fore-aft ground reaction force during braking and propulsive times, respectively, were calculated^33,34^.

#### Vertical stiffness

The vertical oscillation of the whole-body COM as function of time during ${t}_{c}$ was obtained following the method proposed by Cavagna^35^, i.e., by double integration of the vertical acceleration, which permitted to calculate the vertical displacement of the whole-body COM during ${t}_{c}$. Then, vertical stiffness was calculated as the ratio of ${F}_{v,\text{max}}$ over that vertical displacement.

#### Mechanical work

The positive potential, kinetic, and external works were computed [see Cavagna et al.^22^ and Cavagna^35^ for more details]. Briefly, the positive potential work was obtained from the potential energy, itself calculated from the vertical displacement of the whole-body COM, which was obtained from double integration of the vertical acceleration^35^. The positive kinetic work was obtained from the (total) kinetic energy, itself calculated from the 3D velocities of the whole-body COM, which was obtained from single integration of the 3D accelerations^35^. The positive external work was obtained from the external energy, i.e., the sum of the potential and kinetic energy^22,35^. In addition, the positive internal energy (${W}_{\text{int}}$) was estimated using the equation from Nardello et al.^36^, i.e.,

3 $${W}_{\text{int}}=0.08 \text{ SF s} \left[1+ {\left(\frac{\text{DF}}{1-\text{DF}}\right)}^{2}\right],$$

where $\text{s}$ denotes the running speed, DF is the duty factor, and SF is the stride frequency. Finally, the positive internal and external works were summed to give the positive total work. Mechanical work is given in J/kg/m. Data analysis was performed using Python (version 3.7.4, available at http://www.python.org).

### Statistics

Assuming biomechanical differences with moderate effect sizes (~ 0.5), an α error of 0.05, and a power of 0.8, sample size calculations resulted in the requirement of 12 participants^37^. All data are presented as mean ± standard deviation. After having inspected residual plots and having observed no obvious deviations from homoscedasticity and normality, the effect of the time stride selection and percent of the participants’ peak aerobic speed (%PS) on the biomechanical variables were investigated using two-way [(start, mid, end stride selection) x (90, 100, 110, 120%PS)] repeated measures ANOVA (RM-ANOVA) with correction for sphericity if sphericity was violated based on the results of the Mauchly’s test for sphericity and employing Holm corrections for pairwise post hoc comparisons. Even if a statistically significant %PS effect was reported, pairwise post hoc comparisons were not investigated as they did not constitute the main purpose of this study. Similarly, as for a statistically significant interaction effect, only the pairwise post hoc comparisons within a given %PS were investigated. In addition, the number of vertical impact peaks obtained at the start, mid, and end of the four exhaustive runs were compared using Chi-squared (${\upchi }^{2}$) tests. Statistical analysis was performed using Jamovi (version 1.6.18, retrieved from https://www.jamovi.org) with a level of significance set at *P* ≤ 0.05.

## Results

Time to exhaustion, and similarly for the timings of the strides selected at the mid and end, reduced by a factor of 10 with increasing the running speed from 90 to 120% of the participant’s PS while RPE was constant and averaged to 19.0 ± 0.2 over the four exhaustive runs (Table 1). RPE at the end of the maximal incremental aerobic test was 19.6 ± 0.5.

**Figure 1:**
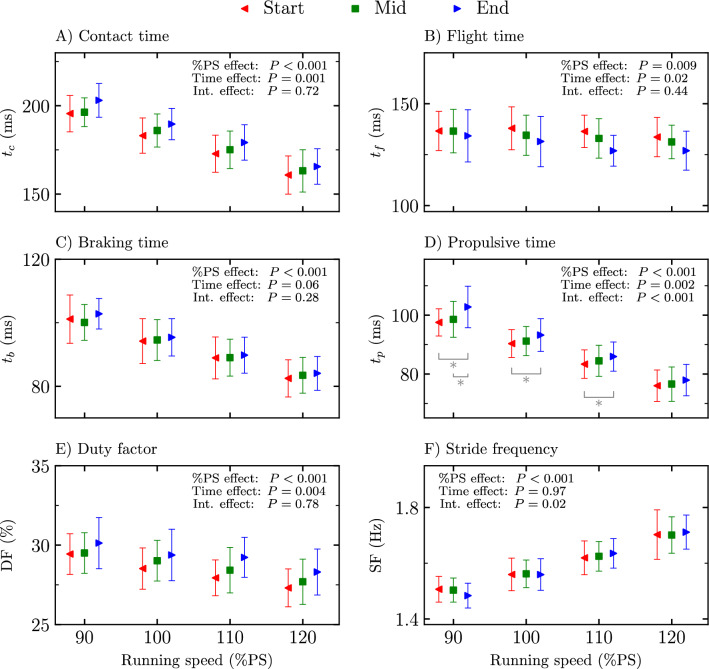
Temporal parameters (**A**) contact time (${t}_{c}$), (**B**) flight time (${t}_{f}$), (**C**) braking time (${t}_{b}$), (**D**) propulsive time (${t}_{p}$), (**E**) duty factor (DF), and (**F**) stride frequency (SF) at the start, mid, and end of the four exhaustive runs, i.e., 90, 100, 110, and 120% of peak aerobic speed (PS) of the maximal incremental aerobic test. Percentage of peak aerobic speed effect (%PS effect), time stride selection effect (time effect), and interaction effect (int. effect) given by the two-way repeated measures ANOVA are depicted. *Significant difference (*P* ≤ 0.05) as reported by Holm post hoc tests and only represented for the pairwise comparisons within a given %PS.

Temporal variables (${t}_{c}$, ${t}_{f},$ braking time, propulsive time, duty factor, and stride frequency) at the start, mid, and end of the four exhaustive runs are depicted in Fig. 1. A significant %PS effect was reported for all temporal variables (*P* ≤ 0.009; *F* ≥ 4.51). A time stride selection effect was obtained for ${t}_{c}$, ${t}_{f},$ propulsive time, and duty factor (*P* ≤ 0.02; *F* ≥ 6.67). Holm post hoc tests revealed a statistically longer ${t}_{c}$, propulsive time, and higher duty factor at end than at start and mid (*P* ≤ 0.02; *t* ≥ − 2.81) and shorter ${t}_{f}$ at end than at start (*P* = 0.004; *t* = 3.61). A significant interaction effect was reported for propulsive time and stride frequency (*P* ≤ 0.02; *F* ≥ 3.54), leading to a longer propulsive time at end than at start for 90, 100, and 110%PS and at end than at mid for 90%PS (*P* ≤ 0.02; *t* ≥ − 3.19) while no pairwise post hoc comparison within a given %PS was statistically significant for stride frequency.

**Figure 2:**
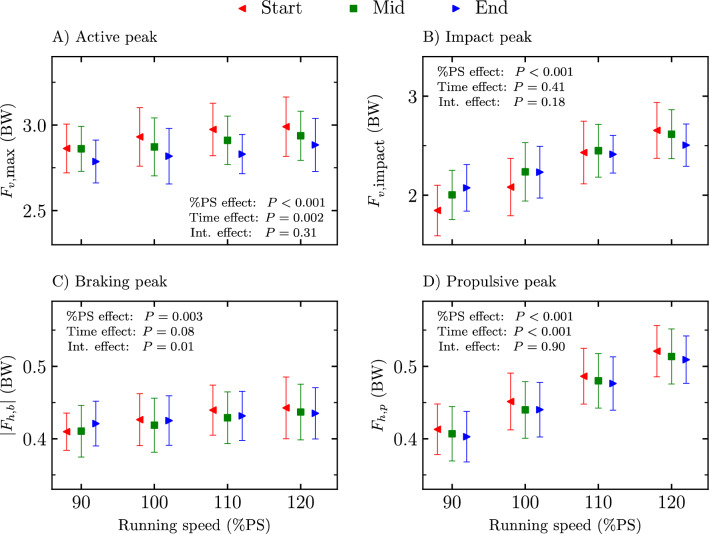
Peaks on the ground reaction force (**A**) active peak (${F}_{v,\text{max}}$), (**B**) impact peak (${F}_{v,\text{impact}}$), (**C**) absolute braking peak ($|{F}_{h,b}|$), and (**D**) propulsive peak (${F}_{h,p}$) at the start, mid, and end of the four exhaustive runs, i.e., 90, 100, 110, and 120% of peak aerobic speed (PS) of the maximal incremental aerobic test. Percentage of peak aerobic speed effect (%PS effect), time stride selection effect (time effect), and interaction effect (int. effect) given by the two-way repeated measures ANOVA are depicted. No pairwise post hoc comparison within a given %PS was statistically significant for the statistically significant interaction effect obtained for ${|F}_{h,b}$|.

Peaks on the ground reaction force (${F}_{v,\text{max}}$, ${F}_{v,\text{impact}}$, braking peak force, and propulsive peak force) at the start, mid, and end of the four exhaustive runs are depicted in Fig. 2. A significant %PS effect was reported for the four variables (*P* ≤ 0.003; *F* ≥ 3.69). A time stride selection effect was obtained for ${F}_{v,\text{max}}$ and propulsive peak force (*P* ≤ 0.002; *F* ≥ 14.49). Holm post hoc tests revealed a statistically smaller ${F}_{v,\text{max}}$ at end than at start and mid and at mid than at start (*P* ≤ 0.04; *t* ≥ 2.36) as well as a statistically smaller propulsive peak force at end than at start and at mid than at start (*P* = 0.004; *t* ≥ 3.96). A significant interaction effect was reported for the braking peak force (*P* = 0.01; *F* ≥ 2.95) but no pairwise post hoc comparison within a given %PS was statistically significant.

The number of vertical impact peaks as well as the number of runners who depicted at least one vertical impact peak are shown in Table 2. The distribution of the number of vertical impact peaks among the start, mid, and end of the four exhaustive runs were statistically significant, as reported by the ${\upchi }^{2}$ test (*P* ≤ 0.04; ${\upchi }^{2}$ ≥ 6.40), with the number of vertical impact peaks at end being greater than those at mid and those at mid being greater than those at start.

**Table 2:** Counts of impact peaks obtained for the ten strides recorded during the start, mid, and end of the four exhaustive runs as well the number of runners depicting at least one impact peak.

Running speed (%PS)	90		100		110		120	
	Impact peaks	Number of runners	Impact peaks	Number of runners	Impact peaks	Number of runners	Impact peaks	Number of runners
Start	17 (7)	4 (31)	34 (13)	5 (38)	60 (23)	10 (77)	90 (35)	10 (77)
Mid	54 (21)	9 (69)	92 (35)	12 (92)	101 (39)	10 (77)	106 (41)	13 (100)
End	129 (50)	12 (92)	133 (51)	11 (85)	158 (61)	13 (100)	127 (49)	12 (92)

Values in parenthesis indicate the corresponding percentages computed over 260 steps and 13 runners. The exhaustive runs were performed at 90, 100, 110, and 120% of the participant’s peak aerobic speed (PS). Chi-squared tests reported that the distribution of the impact peaks among the start, mid, and end were statistically significant for the four exhaustive runs (*P* ≤ 0.04; ${\upchi }^{2}$ ≥ 6.40), with the counts of impact peaks at end being greater than those at mid and those at mid being greater than those at start.

**Figure 3:**
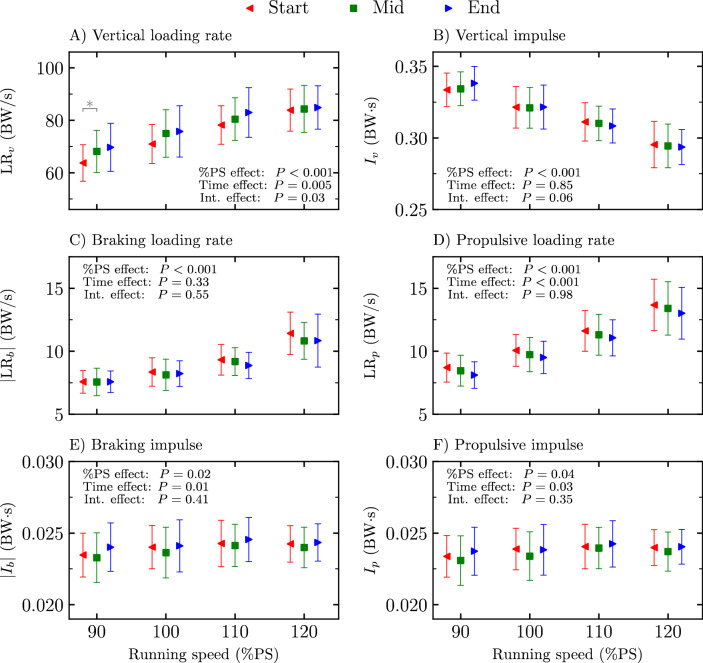
Loading rates and impulses (**A**) vertical loading rate (${\text{LR}}_{v}$), (**B**) vertical impulse (${I}_{v}$), (**C**) absolute braking loading rate (${|\text{LR}}_{b}$|), (**D**) propulsive loading rate (${\text{LR}}_{p}$), (**E**) absolute braking impulse ($|{I}_{b}|$), and (**F**) propulsive impulse (${I}_{p}$) at the start, mid, and end of the four exhaustive runs, i.e., 90, 100, 110, and 120% of peak aerobic speed (PS) of the maximal incremental aerobic test. Percentage of peak aerobic speed effect (%PS effect), time stride selection effect (time effect), and interaction effect (int. effect) given by the two-way repeated measures ANOVA are depicted. *Significant difference (*P* ≤ 0.05) as reported by Holm post hoc tests and only represented for the pairwise comparisons within a given %PS.

Loading rates and impulses (vertical, braking, and propulsive) at the start, mid, and end of the four exhaustive runs are depicted in Fig. 3. A significant %PS effect was reported for all loading rates and impulses (*P* ≤ 0.04; *F* ≥ 3.69). A time stride selection effect was obtained for vertical and propulsive loading rates and braking and propulsive impulses (*P* ≤ 0.03; *F* ≥ 4.18). Holm post hoc tests revealed a statistically smaller vertical loading rate at start than at mid and end (*P* ≤ 0.01; *t* ≥  − 3.30), a statistically smaller propulsive loading rate at end than at start and mid and at mid than at start (*P* ≤ 0.008; *t* ≥ 3.19), as well as statistically larger braking and propulsive impulses at end than at mid (*P* ≤ 0.02; *t* ≥  − 3.25). A significant interaction effect was reported for the vertical loading rate (*P* = 0.03; *F* ≥ 3.52), leading to a larger vertical loading rate at mid than at start for 90%PS (*P* = 0.05; *t* =  − 4.37).

**Figure 4:**
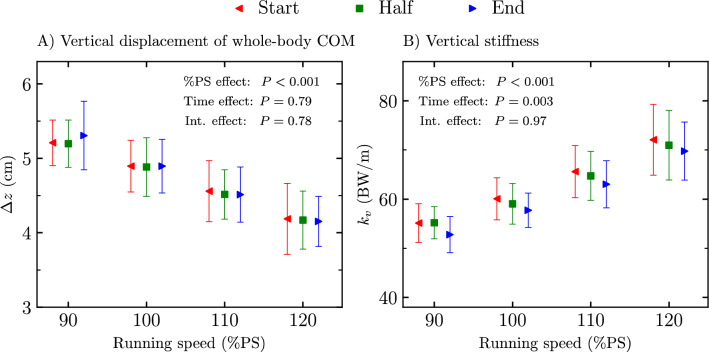
(**A**) Vertical displacement of the whole-body center of mass (COM) during ground contact time ($\Delta z$) and (**B**) vertical stiffness (${k}_{v}$) at the start, mid, and end of the four exhaustive runs, i.e., 90, 100, 110, and 120% of peak aerobic speed (PS) of the maximal incremental aerobic test. Percentage of peak aerobic speed effect (%PS effect), time stride selection effect (time effect), and interaction effect (int. effect) given by the two-way repeated measures ANOVA are depicted.

The vertical oscillation of the whole-body COM and vertical stiffness at the start, mid, and end of the four exhaustive runs are depicted in Fig. 4. A significant %PS effect was reported for both the vertical oscillation and vertical stiffness (*P* < 0.001; *F* ≥ 77.10). No time stride selection effect was obtained for the vertical oscillation (*P* = 0.79; *F* = 0.13) while a time stride selection effect was obtained for the vertical stiffness (*P* = 0.003; *F* = 7.54). Holm post hoc tests revealed statistically smaller vertical stiffness at end than at start and mid (*P* ≤ 0.03; *t* ≥  − 3.08). No significant interaction effect was reported for both the vertical oscillation and vertical stiffness (*P* ≥ 0.78; *F* ≤ 0.54).

**Figure 5:**
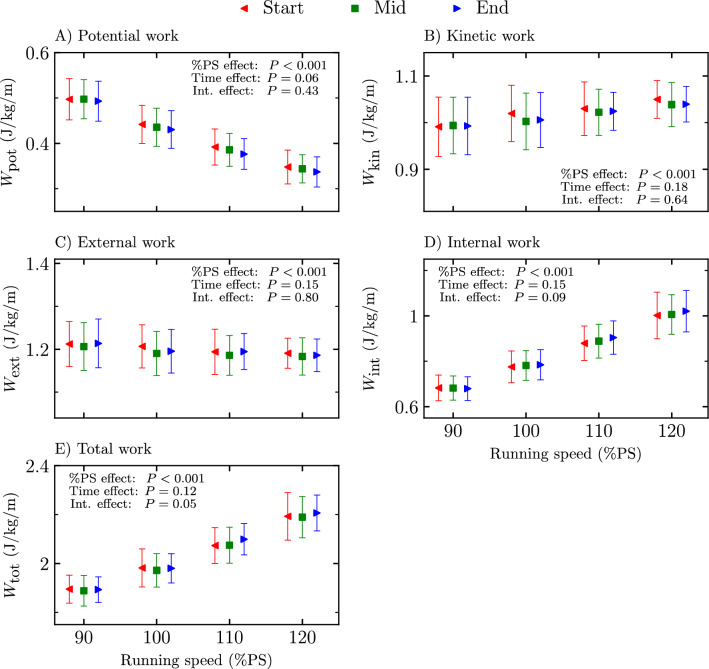
Mechanical work (**A**) potential work (*W*_pot_), (**B**) kinetic work (*W*_kin_), (**C**) external work (*W*_ext_), (**D**) internal work (*W*_int_), and (**E**) total work (*W*_tot_) at the start, mid, and end of the four exhaustive runs, i.e., 90, 100, 110, and 120% of peak aerobic speed (PS) of the maximal incremental aerobic test. Percentage of peak aerobic speed effect (%PS effect), time stride selection effect (time effect), and interaction effect (int. effect) given by the two-way repeated measures ANOVA are depicted. No pairwise post hoc comparison within a given %PS was statistically significant for the statistically significant interaction effect obtained for* W*_tot_.

Mechanical work (positive total, internal, external, potential, and kinetic) at the start, mid, and end of the four exhaustive runs are depicted in Fig. 5. A significant %PS effect was reported for all mechanical work (*P* < 0.001; *F* ≥ 7.71). No time stride selection effect was obtained for any mechanical work variable (*P* ≥ 0.06; *F* ≤ 1.98) while a significant interaction effect was reported for the positive total mechanical work (*P* = 0.05; *F* = 2.27). However, no pairwise post hoc comparison within a given %PS was statistically significant.

## Discussion

**Figure 6:**
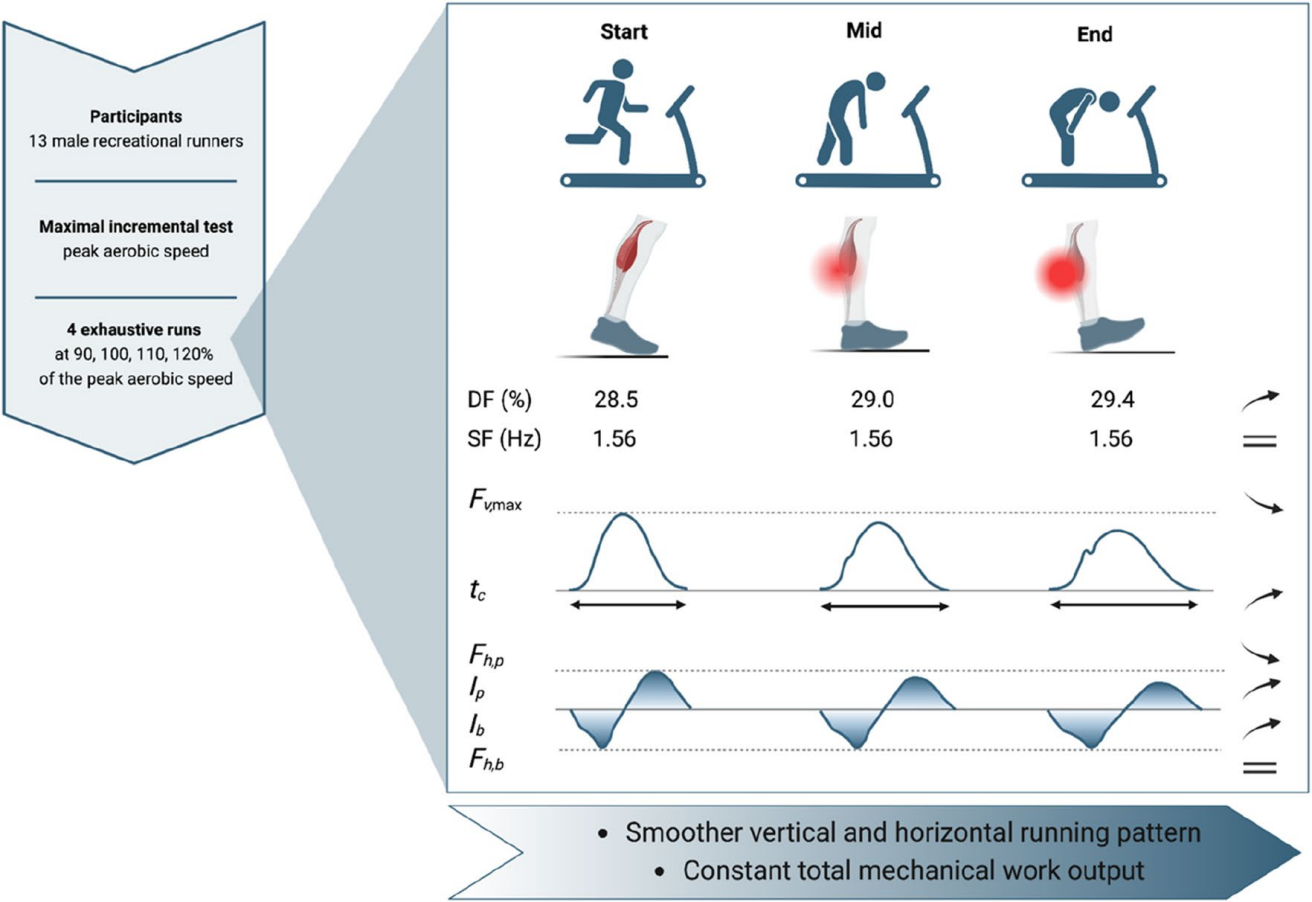
Main biomechanical adaptations measured at start, mid, and end of exhaustive runs at 90 to 120% of peak aerobic speed. These adaptations were similar among the four tested running speeds. The smaller peak vertical ground reaction force (${F}_{v,\text{max}}$), higher duty factor (DF) due to longer contact time (*t*_*c*_) and unchanged stride frequency (SF), and higher prevalence of rearfoot strikers (reduced activation of the plantar flexor muscle–tendon units) towards the end of the exhaustive runs suggest a tendency towards a smoother vertical running pattern with exhaustion. The larger braking (*I*_*b*_) and propulsive (*I*_*p*_) impulses and lower propulsive peak force (*F*_*h,p*_) also suggest a smoother horizontal running pattern with exhaustion though the braking peak force (*F*_*h,b*_) did not change. Despite these changes of running pattern with exhaustion, there were no changes in either gesture speed (unchanged SF) or positive mechanical work, advocating that runners unconsciously organize themselves to maintain a constant total mechanical work output. DF and SF values are those corresponding to the 100% peak aerobic speed running trial. Created with BioRender.com.

In accordance with the first hypothesis, a longer ${t}_{c}$ and a shorter ${t}_{f}$ were found with exhaustion. However, in contrast with this hypothesis, there was no change in stride frequency. Additionally, in line with the second hypothesis, ${F}_{v,\text{max}}$ decreases throughout the time course of the exhaustive runs, but in contrast with this hypothesis, the vertical loading rate and stiffness increased and decreased, respectively. Finally, internal and external mechanical work stayed constant throughout the time trials to exhaustion, with no change in potential and kinetic mechanical works. The change of running biomechanics with fatigue (evaluated at start, mid, and end) was similar among the four tested running speeds (90 to 120%PS) as suggested by the fact that almost all post hoc comparisons within a given %PS (interaction effect) were not significative. Main biomechanical adaptations measured at start, mid, and end of the exhaustive runs are reported in Fig. 6.

A significant increase in ${t}_{c}$ and a significant decrease in ${t}_{f}$ with exhaustion were reported (Fig. 1). Though conflicting evidence exists with respect to changes in ${t}_{c}$ and ${t}_{f}$ with exhaustion, the present study corroborates previously published results obtained for both ${t}_{c}$^4–6,13,38^ and ${t}_{f}$^5,6,13^ at 90 to 100%${s}_{{\dot{\text{V}}}{{\text{O}}}_{2}\text{max}}$. However, the present results contradict the continuous decrease in ${t}_{c}$ and increase in ${t}_{f}$ reported by Hanley and Mohan^7^ during a 10-km treadmill run performed at 103% of season’s best time. The authors attributed these findings to a treadmill effect and proposed that athletes altered their running pattern so that more time was spent airborne to allow the treadmill to pass under them. However, the present study also used a treadmill. Moreover, (1) the running speed corresponding to the 90%PS running trial used herein was similar to the speed employed by these authors (17.1 ± 1.0 km/h herein vs 17.5 km/h) and (2) the authors already observed a decrease in ${t}_{c}$ and an increase in ${t}_{f}$ at the 5 km mark of their 10-km treadmill run, which is roughly the distance ran herein at 90%PS (4.4 ± 0.7 km, obtained from the 90%PS values and corresponding time to exhaustions). Hence, these discrepancies could not readily be explained. Overall, the present results confirm that ${t}_{c}$ increases and ${t}_{f}$ decreases with exhaustion.

${F}_{v,\text{max}}$ significantly decreased with exhaustion (Fig. 2) for running speeds between 90 to 120%PS, which corroborates the findings of Rabita et al.^13^. These authors observed a ~ 10% decrease in ${F}_{v,\text{max}}$ at 95%${s}_{{\dot{\text{V}}}{{\text{O}}}_{2}\text{max}}$. These authors also observed a ~ 10% decrease in vertical oscillation of the whole-body COM, leading to no change in vertical stiffness with exhaustion, which contradicts the present findings. The present data showed a lack of change in vertical oscillation, leading to a decrease in vertical stiffness (Figs. 1, 4). Sanno et al.^39^ reported similar findings for the vertical oscillation and ${F}_{v,\text{max}}$ between the start and end of a 10 km run with near maximal effort. These authors also reported a redistribution of joint work from the ankle to the hip with exhaustion and attributed the appearance of fatigue to the fact that the proximal joints are less equipped for storage and release of elastic energy than ankle plantar flexors. Hence, the decrease in vertical stiffness with exhaustion, i.e., a global parameter which permits to evaluate the “quality” of the spring-mass model^40^, could partly reflect those previous suggestions.

${F}_{v,\text{max}}$ was shown to be inversely proportional to duty factor under a sine-wave approximation of the vertical ground reaction force^41,42^, which justifies the significant increase of duty factor (Fig. 1) with exhaustion reported herein. The increase in duty factor was due to both an increase in ${t}_{c}$ and a decrease in ${t}_{f}$ (Fig. 1), which corroborates previous results which attributed the increase in duty factor with fatigue to a decrease in ${t}_{f}$^10^. However, the results presented herein contradict previous results which showed that the increase in duty factor with fatigue was due to an increase in stride frequency^10^. Indeed, the present results did not report any change in stride frequency with fatigue (Fig. 1), most likely because the increase in ${t}_{c}$ compensates the decrease in ${t}_{f}$, keeping the stride time (and thus stride frequency) constant with fatigue.

The lack of change in stride frequency reported herein (Fig. 1) could be justified by the unchanged vertical impulse with exhaustion (Fig. 3). Indeed, as vertical momentum is conserved during a running step (Newton’s second law of motion), the vertical impulse is equal to body weight multiplied by step time^43^. Hence, the unchanged vertical impulse with exhaustion reflects no change in stride frequency. These results confirm the previous findings of Hayes and Caplan^5^ and Fourchet et al.^6^ for exhaustive runs at 95 to 100%${s}_{{\dot{\text{V}}}{{\text{O}}}_{2}\text{max}}$. The unchanged stride frequency with fatigue is accompanied by a reorganization of the timings (Fig. 1), with a longer ${t}_{c}$ and shorter ${t}_{f}$ observed with fatigue, and also suggests no change in step length with exhaustion (an increase/decrease in stride frequency is necessarily associated with a decrease/increase in step length during running at constant speed). However, other studies reported both a smaller than or equal to 3% decrease^1,2,4,8^ or a greater than or equal to 2% increase^13^ in stride frequency with fatigue, which are opposed to the present findings. This suggests that running trials to exhaustion at 90 to 120%PS did not modify the *gesture speed* during running (no alteration of stride frequency) but should lead to a muscular fatigue, especially of the muscles providing running propulsion.

The plantar flexor muscle–tendon units may also be characterized by a reduction of their activation during the braking phase^44^ likely due to a transition towards a rearfoot strike pattern [i.e., load-shifting strategy^45^]. In fact, the increase of both the number of runners showing at least one vertical impact peak and the number of vertical impact peaks with exhaustion (Table 2) indicates a transition towards this specific foot strike pattern^46^ corroborating previous findings showing higher prevalence of rearfoot runners at the end of a marathon^11,12^. The present results suggest that this foot-strike pattern transition seemed continuous between the start and end of the running trials. This transition was accompanied with an increase in vertical loading rate with exhaustion (Fig. 3). This result contradicts the lower vertical loading rate observed at the end of ultra-distance runs^9,10,14,15^, which was attributed to the development of protective adjustments leading to a minimization of the overall load applied to the musculoskeletal system. However, this can be explained by the fact that the choice of foot-strike pattern may be task-specific^47^. The authors claimed that running a long distance may require a rearfoot strike pattern to minimize the metabolic cost of running while a more anterior foot-strike pattern may be necessary to run faster. Hence, on the one hand, the runners of the present study seemed to transition from a forefoot to a rearfoot strike pattern with exhaustion, and thus increased their vertical loading rate. On the other hand, ultra-distance runners already adopted a rearfoot strike pattern in the pre-test because they knew they were going to run an ultra-distance and kept their foot-strike pattern while making their global running pattern smoother all along their long-distance run, and thus reduced their vertical loading rate in the post-test compared to the pre-test.

The running pattern seemed to continuously become smoother along the vertical axis with exhaustion, as reported by the transition towards a rearfoot strike pattern and the increase in duty factor (though not significant between start and mid) and decrease in ${F}_{v,\text{max}}$ between the start and end of the running trials (Figs. 1, 2). These results agreed with the tendency towards a smoother vertical running pattern (lower ${F}_{v,\text{max}}$ and vertical loading rate and higher stride frequency and duty factor) observed by Millet et al.^10^ at the end of an ultra-distance run (8500 km) and by others after an ultra-trail 110 to 330 km^9,14,15^. The present study did not observe a decrease in vertical loading rate and increase in stride frequency with exhaustion for the reasons previously explained. The results presented herein further suggest that these different mechanisms to smoothen the running pattern might come from the different types of engendered fatigue. Indeed, Millet et al.^10^ investigated a runner performing a 8500 km run in 161 days while this study investigated time trials to exhaustion at 90 to 120%PS. In both cases, such smoother running pattern is adopted by the runners to decrease the muscle force level during the propulsion phase., i.e., to develop protective adjustments, to decrease the risk to develop a running-related injury.

The longer ${t}_{c}$ with exhaustion was associated with a longer propulsive time but no change in braking time (Fig. 1). This change was associated with a smaller propulsive peak force but no change in braking peak force (Fig. 2), a lower propulsive loading rate but no change in braking loading rate, and larger braking and propulsive impulses (Fig. 3) with exhaustion. Similar findings were previously reported for propulsive and braking peak forces but no change of their corresponding impulses was obtained with exhaustion^13^. The present results further suggest that runners needed more time to reorient the fore-aft component of the ground reaction force signal to counter the previous braking phase in a fatigued state. A longer propulsive time and lower propulsive peak force with exhaustion allow to explain the lower propulsive loading rate obtained herein. Besides, the increase in propulsive impulse with exhaustion could be explained by a more pronounced increase in propulsive time than decrease in propulsive peak force. Hence, since braking and propulsive impulses must be equal because of the constant running speed, the braking impulse must necessarily increase with exhaustion. However, this increase in braking impulse was not reflected in braking time and braking peak force, which could be explained by a cumulative change of both braking time and braking peak force variables, though too small to be statistically significant.

The increase of ${t}_{c}$ with exhaustion was previously attributed to an intrinsic mechanism that allow runners to maintain a constant horizontal impulse despite fatigue development^6,13^. The results of the present study do not exactly support this statement as a significant increase in braking and propulsive impulses was reported with exhaustion (Fig. 3). However, the present results showed that with exhaustion, the braking impulse increased via an increase in braking time with no change in braking peak force. Moreover, the propulsive impulse increased via a larger relative increase in propulsive time than the relative decrease in propulsive peak force with exhaustion (Figs. 1, 2). Hence, this suggests that runners also adopt a smoother running pattern in the anterior–posterior axis with exhaustion. The increase in propulsive time (though not significant between start and mid) and decrease in propulsive peak force with exhaustion (Figs. 1, 2) suggest that the horizontal running pattern seems to continuously become smoother between the start and end of the running trials. The fatigued muscles contributing to propulsion, mostly the plantar flexor muscles^48^, seem to be recruited upon at a lower force level but over a longer period. Similarly, ankle plantar-flexor resistance to fatigue was shown to decrease after an exhaustive run at 95%${s}_{{\dot{\text{V}}}{{\text{O}}}_{2}\text{max}}$^49^. This smoother horizontal running pattern is also reflected in the decrease in propulsive loading rate with exhaustion. Moreover, increasing the braking and propulsive times deteriorates the stretch–shortening cycle^50^, hence further supporting the decrease in vertical stiffness and indirectly the transition towards the usage of more proximal joints which are less spring-mass efficient^39^.

Despite the change of running pattern with exhaustion, there was no change in potential, kinetic, external, internal, and total positive mechanical work (Fig. 5), suggesting that runners organize unconsciously themselves to maintain their mechanical work constant. These findings corroborate those of Avogadro et al.^4^ obtained between the third and last minute of an exhaustive run at 90%${s}_{{\dot{\text{V}}}{{\text{O}}}_{2}\text{max}}$. The lack of change in external mechanical work with fatigue allows confirming that the increase in ${\dot{\text{V}}}{{\text{O}}}_{2}$ is not mediated by a change in mechanical work^1,4^. However, though Borrani et al.^1^ reported a constant external mechanical work, these authors obtained an increase in potential mechanical work and a decrease in kinetic mechanical work as well as a decrease in internal mechanical work, due to a decrease of both stride frequency and ${t}_{\text{c}}$, between the start and the end of an exhaustive run at a speed corresponding to 95%${s}_{{\dot{\text{V}}}{{\text{O}}}_{2}\text{max}}$, which contradicts our findings. Similarly, the present results disagreed with the lower internal mechanical work and larger external mechanical work reported at the end than at the beginning of an exhaustive run performed at a speed corresponding to the participants’ personal record over 3000 m^2^. These discrepancies may be due to methodological differences associated with the device used to assess the mechanical power output, i.e., kinematic arm vs instrumented treadmill. Nevertheless, the present study depicted no effect of the time stride selection when calculating positive mechanical work from 90 to 120%PS. Thus, these results further extended the knowledge of exhaustion on work calculations, especially at 110 and 120%PS, by showing that mechanical work stayed constant with exhaustion.

A few limitations to the present study are worth noting. The participant should have completed five experimental sessions interspersed by at least 2 days, which could question the fact that they gave their maximum during each time trial to exhaustion. Moreover, no test–retest repeatability of time to exhaustion has been performed. However, even if repeatability was shown to vary by up to 15%^51^ and familiarization was shown to increase reliability^52^, this choice was made to avoid further increasing the number of experimental sessions because this would tends to be unpractical for the participants. Furthermore, there was no isotime measurement, which makes it not possible to compare the effect of running speed independently of the duration, though this was not the main purpose of this study. Additionally, the external mechanical work was computed from the displacement and velocity of the COM and not using a segmental analysis. Hence, they may have been a redistribution of work from one joint to another that could not be assessed herein. The internal mechanical work was estimated using the equation from Nardello et al.^36^, which used running speeds up to 3.06 m/s. Hence, this model has not been verified using the speeds used in the present manuscript. Finally, as no EMG and strength measures were performed, the suggestion of a change in timing in muscle recruitment, muscular fatigue, and load shifting strategy should be further investigated.

## Conclusion

To conclude, the change of running biomechanics (spatiotemporal and kinetic variables) with fatigue (evaluated at start, mid, and end) was similar among the four tested running speeds (90 to 120%PS). The smaller ${F}_{v,\text{max}}$, higher duty factor, and higher prevalence of rearfoot strikers towards the end of the exhaustive runs suggest a tendency towards a smoother vertical running pattern with exhaustion. The longer propulsion time (and propulsive impulse) and corresponding lower propulsive peak force and loading rate also suggest a smoother horizontal running pattern with exhaustion. The transition towards a smoother vertical and horizontal running pattern with exhaustion seemed continuous between the start and end of the running trials and could be adopted by the runners to decrease the muscle force level during the propulsion phase. Despite these changes of running pattern with exhaustion, there were no changes in either gesture speed (unchanged stride frequency) or positive mechanical work, advocating that runners unconsciously organize themselves to maintain a constant total mechanical work output.

## Data Availability

The datasets used and/or analyzed during the present study are available from the corresponding author on reasonable request.
